# The Distributions and Dependences of 3D Particle Morphology Characteristics for Crushed and Natural Sands by X-Ray uCT Investigations

**DOI:** 10.3390/ma17235805

**Published:** 2024-11-27

**Authors:** Hao Yang, Xu Li, Junhui Zhang, Zhengbo Hu, Shengnan Li

**Affiliations:** 1 Key Laboratory of Highway Engineering of Ministry of Education, Changsha University of Science & Technology, Changsha 410114, China; 2 School of Traffic and Transportation Engineering, Changsha University of Science and Technology, Changsha 410114, China; 3 School of Architectural Engineering, Hunan Institute of Engineering, Xiangtan 411104, China

**Keywords:** 3D shape characteristics, ellipsoidal degree, microscale size properties, specific surface, X-ray uCT, crushed and natural sands

## Abstract

The morphology of an individual particulate refers to its shape characteristics and size properties, which both play important roles for granular matter in physics, mechanics, chemistry, and biology. In this study, ellipsoidality is defined as a 3D shape index for evaluating particle roundness, and an explicit calculation method is applied. The dependences of 3D shape characteristics (aspect ratios, sphericity, and ellipsoidal degree) on particle size (ranges from 0.063 mm to 5.0 mm) are adequately investigated with the X-ray micro-computed microtomography (uCT) imaging for hundreds of thousands of particles of crushed and natural sands. This study focuses on comparing and evaluating the specific surface area and equivalent diameter, suggesting that particle segregation and changes in surface area may explain the strong dependence of particle shape on size. The correlation between different shape metrics was analyzed by comparing crushed sand with natural sand to provide theoretical support for material filling and mechanical behaviour. The significant differences in the microscale particle size indexes of different sands by single grading are used to provide data references for further analyses of the effect of material microscale on material properties in future discrete element particle simulations.

## 1. Introduction

Recent research has revealed many findings on the impact of particle size, shape, and their gradations on the mechanical behaviour of particulate materials. The morphology of one individual particulate refers to its shape characteristics and size properties, which both play important roles for granular matter in physics, chemistry, biology, and mechanics.

For the study of granular matter in physics, jamming, packing, and segregation phenomena play an important role. Jaeger (2015) [1] suggested that jamming does not rely on temperature like ordinary phase transitions but instead on local geometric constraints. The dependence of local geometric constraints is mainly on the particle characteristics, which are particle size, shape, and their gradations. A study by Wei D et al. (2021) [2] adopts the combined finite-discrete element method (FDEM) to investigate single-particle crushing behaviour. Meng et al. (2017) [3] found that the dense random packings of identical intersecting spherocylinders with central symmetry are numerically simulated through an analytical model and the relaxation algorithm. Particle shape-driven segregation in particulate mixtures has received limited attention in the literature, both experimentally and via simulations. Hu Z et al. (2024) [4] proposed a new method that can precisely control the degree of wear and more accurately mimic natural processes. The investigation by Baur and Huang (2017) [5] provided an example where the collective behaviour of granular matter was tuned by the shape of individual particles. For the study of granular matter in chemistry and biology, particle characteristics also play significant roles ([6,7]); for example, effective thermal conductivity is one of the most important thermal properties of packed granular materials and is significantly affected by particle properties.

For the study of the mechanics of granular matter, mechanical properties mainly depend on the particle size, shape, and their gradations. Santamarina and Cho (2004) [8] suggested that the systematic assessment of particle shape will lead to a better understanding of sand behaviour. A potential concern is the preservation of particle properties with scaling; for example, particle shape has been shown to influence the bulk density, stiffness, and strength of natural and crushed sands [9]. Wang H et al. [10] believed that different particle shapes affected the inherent properties of particle materials. Particle shape affects the mechanical behaviour of soils, including small-strain stiffness, oedometric stiffness, strength, critical state, dilation and peak friction angle, residual friction angle, drained and undrained strength anisotropy, monotonic and cyclic liquefaction, volume change during shear, localization, capillary force of unsaturation, conduction and diffusion, packing, shape sorting and segregation of granular flow, and filters for fluid-related phenomena ([10,11,12,13,14,15,16,17]).

Altuhafi and Coop (2011) [18] examined the changes to some important particle size and characteristics such as 2D particle shape and particle surface roughness in an attempt to relate soil behaviour to the nature of microscopic particle damage during yielding. Cheng Z et al. [19] used the interparticle contact evolution data measured by particle kinematics and X-ray microtomography (μCT) to estimate the contact force chains of uniformly sized spherical granular materials under triaxial compression. Wang L et al. [17] investigated the influence of particle shape, particle size, and particle size distribution on the gravity behaviour of crushed ore in sublevel caving. However, the distributions of 3D particle shape, individual micro-size characteristics [20], and the dependence of morphology on particle size for crushed and natural sands are not clear in the current research. This study investigates the correlations between the physical phenomenon for granular matter and the reason why the dependence of particle shape on particle size is notable.

Existing studies describe particle morphology mostly in terms of the aspect ratio and sphericity, and the average values of sphericity and roundness with two-dimensional shape information are usually used as indicators of the regularity of the particles, while the study of three-dimensional shape indicators is still flawed. In this study, ellipticity is introduced as a three-dimensional shape metric to refine the information describing particle morphology. This study will investigate the trends of the dependence of 3D particle true shapes (aspect ratio, sphericity, and ellipsoidal degree) on particle size (both macro-size index and micro-size index) of natural sands and crushed sands (ranges from 5.0 mm to 0.063 mm) for the first time by X-ray microtomography. Through analyses of the distribution patterns for particle shapes and sizes of natural and crushed sands, the trends of the dependences of the true shape of 3D particles on particle size will be uncovered and the reasons of this phenomenon will be explored. The particle shape characteristics of different types of sand and the distribution characteristics under full-scale mono-gradation are obtained, filling the gap in micro-scale particle distribution patterns. This study reveals the dependence of three-dimensional particle shapes on particle size, investigates the relationships and objective laws among different particle shape indices, and provides a micro-scale basis for further research on the macroscopic properties of materials. Additionally, this study offers data support for particle shape simulation across various gradations.

## 2. Particle Morphology

Shape indexes (both in 2D and in 3D) can be categorized as two kinds of concepts, i.e., explicit shape indexes and implicit shape indexes, based on the types of algorithms for the calculations of definitions. The aspect ratio and sphericity are typical examples of explicit shape indices. The aspect ratios are the ratios between different the main Feret diameters. The sphericity is the ratio between the surface area of the sphere with the equivalent volume and the surface area of the particle. However, other particle shape indexes, such as convexity and roundness, are defined with implicit geometry. They are not refined calculations with high efficiency and will obtain quite significantly different results by different approximation algorithms due to the definitions without explicit physical meanings, especially for 3D implicit shape indexes [20]. The 3D particle shape indexes are much better than 2D ones, especially compared to those obtained using the 2D visual inspection method because the information of 2D is very stochastic.

Particle imaging has traditionally been based on 2D projections, but contemporary imaging now uses digital cameras for 2D and laser scanning or computer tomography (CT) scanning for 3D. A method of defining form, based on the smallest dimension of a scalene ellipsoid with the same volume as any given particle, has been proposed, and a method of measuring it using static imaging has been described [21]. It has been demonstrated that the SEES approach to determining S, I, and L is reasonably consistent with a two-view approach for the ballast investigated and has the advantage of being less time-consuming.

A new measure of roundness (termed ellipseness) has been introduced. This relates the perimeter of the particle to the perimeter of an idealized elliptical particle of the same area and can be determined automatically using appropriate imaging and analysis software [22]. Currently, there is no 3D definition for roundness with explicit calculation.

### 2.1. Definition of Ellipsoidal Degree

Ellipseness is a 2D concept, was proposed for evaluating the roundness of a railway ballast by Le Pen et al. (2013) [22]. Many of the current definitions of roundness are implicit shape indexes, while ellipseness is the only one for roundness with an explicit definition. Explicit definitions show more advantages in accurate and efficient calculations than implicit definitions. However, ellipseness is a 2D definition for the roundness of coarse grains, such as railway ballast, which is proposed for 2D manual imaging experiments. Based on the ellipseness, a 3D definition called the ellipsoidal degree is proposed in this study for CT imaging experiments with more accurate and complete information on particles.

The ellipsoidal degree is a 3D concept and is defined as the ratio between the surface area of the scalene ellipsoid with the equivalent volume and the surface area of the particle. The equation for the ellipsoidal degree is shown below.

(1) $$ED=\frac{A_{ellipsoid\;with\;the\;equivalent\;volume\;V}}{A_{particulate}}$$

where *ED* is the ellipsoidal degree of a particle, *A* is the surface area of a particle, and *V* is the volume of a particle.

The three main dimensions of the ellipsoid are determined by the two main Feret diameters and the same volume value of the researched particle. It is an explicit shape index defined with the main Feret diameters, volume, and surface area of particles. When two of the three main Feret diameters are known and regarded as two of the main dimensions of the ellipsoid, the third dimension of the ellipsoid can be calculated with the equivalent volume value of the particle. So, there are three descriptors of ellipsoidal degrees in total. ED1 can be obtained by the known longest and medium Feret diameters. ED2 can be obtained by the known medium and shortest Feret diameters. ED3 can be obtained by the known longest and shortest Feret diameters. ED is taken as the average value of these three descriptors, ED1, ED2, and ED3.

The mathematical function of the ellipsoid is

(2) $$\frac{x^{2}}{a^{2}}+\frac{y^{2}}{b^{2}}+\frac{z^{2}}{c^{2}}=1\;(a>0,\;b>0,\;c>0)$$

where *a*, *b*, and *c* are the size of the ellipsoid, which is called the long half-axis, the middle half-axis, and the short half-axis of the ellipsoid, respectively.

The volume calculation equation of the ellipsoid is

(3) $$V=\frac{4\pi abc}{3}$$

The surface area calculation equation of the ellipsoid is

(4) $$A=4\pi {\left(\frac{a^{p}b^{p}+b^{p}c^{p}+c^{p}a^{p}}{3}\right)}^{1/p}$$

where *p* ≈ 1.6 (Clayton et al., 2009 [21]).

### 2.2. Advantage of Ellipsoidal Degree

The ellipsoid is a more shaped analogue with natural sands, which range from rounded to elongated shapes. Also, the ellipsoidal degree is an independent index for distinguishing the feature of aspect ratios. However, the roundness proposed by Wadell (1932) [23] highly depends on the aspect ratio of the particle. For both the sphere and the ellipsoid, their ellipsoidal degrees are the same. However, the aspect ratio of the sphere is one, while the aspect ratio of the ellipsoid is less than one.

The comparison of the ellipsoidal degrees and the traditional roundness of the typical 3D geometries is shown in Table 1. For the definition of roundness by Wadell, the corner is defined as every such part of the outline of an area (projection area), which has a radius of curvature equal to or less than the radius of curvature of the maximum inscribed circle of the same area. However, there is no specific value of roundness for the triangular pyramid and the cuboid because there is no value of the radius of the curvature for some sharp corners. Also, there are infinite corners for a natural particle. Thus, the roundness by Wadell is not suitable for the application of granular assembly with lots of particles because the value of the roundness of one particle is uncertain using different algorithms.

The ellipsoidal degree in this study is the first definition of the 3D roundness of particles with an explicit calculation. It overcomes all the disadvantages of the method of roundness by Wadell.

## 3. Distributions of Shape Indexes

The Weibull distribution was first applied by Rosin and Rammler (1933) [24] to describe particle size distributions. It is widely used in mineral processing to describe particle size distributions in comminution processes.

The three-parameter Weibull distribution is a form of Weibull distribution. It is determined by three parameters: shape, scale (range), and position. The shape parameter is the most important parameter, which determines the basic shape of the distribution density curve. The scale parameter can enlarge or reduce the function of the curve, but it does not affect the shape of the distribution. It can also be used as an approximation of many other distributions; for example, the shape parameter can be set to the appropriate value to approximate the normal distribution.

The shape properties of sands approximate the skewed normal distribution, and the Weibull distribution function is used here to obtain the approximate the normal distribution. The probability distribution function (PDF) and cumulative distribution function (CDF), mean value, and variance in four kinds of natural or crushed sands are compared and analyzed.

The equation of the three-parameter Weibull PDF is

(5) $$PDF=f\left(x;k,\lambda ,\theta \right)=\frac{k}{\lambda }{\left(\frac{x-\theta }{\lambda }\right)}^{k-1}e^{{-\left(\frac{x-\theta }{\lambda }\right)}^{k}}$$

for *x* ≥ *θ* and *f*(*x*; *k*, *λ*, *θ*) = 0 for *x* < *θ*.

The Weibull cumulative distribution function (CDF) is given by

(6) $$CDF=f\left(x;k,\lambda ,\theta \right)=1-e^{{-\left(\frac{x-\theta }{\lambda }\right)}^{k}}$$

where *k* > 0 is the shape parameter, *λ* > 0 is the scale parameter, and *θ* is the location parameter of the distribution. When *θ* = 0, this reduces to the two-parameter distribution.

The confidence level alpha is set to 0.05 with the maximum likelihood method for parameter estimation (Smith & Naylor 1987) [25].

The particle characteristics (shape and individual size) of four kinds of silica sands are investigated by X-ray uCT in this study. Angular quartz sand (AQS) is a kind of standard quartz sand obtained through mechanical crushed processing in the factory. Zhujiang river sand (ZRS) is a kind of quartz sand from Zhujiang river (Pearl Sea), south of Guangdong province, China. Rounded quartz sand (RQS) is a kind of quartz sand that consists of variational shapes of particles from Zhuhai, south of Guangdong province, China. Leighton Buzzard sand (LBS) is a silica sand that consists of strong and regular highly spherical particles from the UK.

### 3.1. Distributions of Aspect Ratios

**Figure 1:**
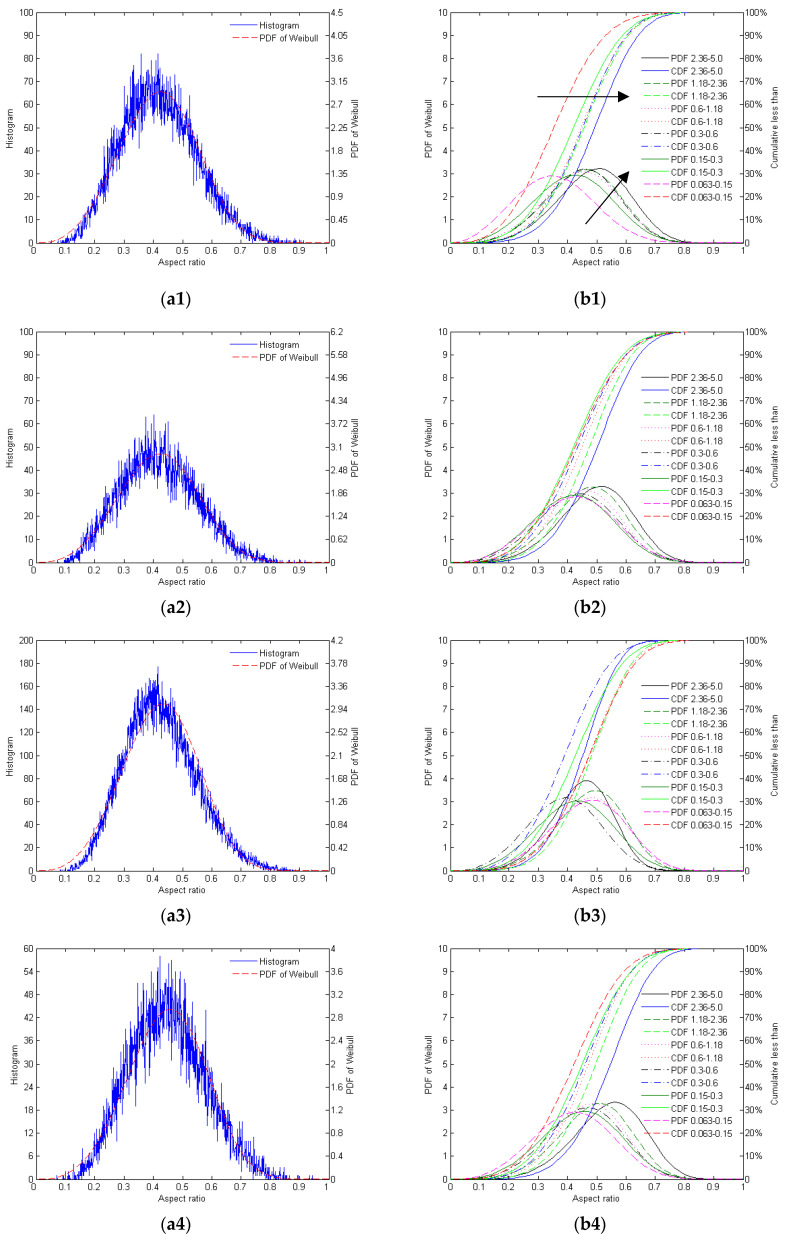
The distribution of aspect ratios of the different mono-gradations of AQS (**a1**,**b1**), ZRS (**a2**,**b2**), RQS (**a3**,**b3**), and LBS (**a4**,**b4**): (**a**) histogram for the mono-gradation ‘0.15–0.3’ and its Weibull PDF; (**b**) the PDF and CDF of Weibull distribution of different mono-gradations.

The distribution of the aspect ratios of the different mono-gradations of crushed and natural sands is shown in Figure 1.

As shown in Figure 1, the particle morphology of AQS is relatively concentrated, with the distribution of the peak aspect ratios being between 0.3 and 0.5. However, due to the crushing process, some particles exhibit irregular shapes, resulting in a relatively broad distribution, which fits the Weibull distribution well. ZRS exhibits diverse particle morphologies, with a dispersed aspect ratio distribution. The main peak is also between 0.3 and 0.5, but there is still a significant distribution at higher aspect ratios (>0.6), indicating complex shapes and that the Weibull fit shows some deviation in the higher range. RQS has more regular and concentrated particle morphologies, with aspect ratios primarily distributed between 0.3 and 0.5, fitting the Weibull distribution very well, suggesting a high consistency in particle shapes. LBS exhibits the most regular particle morphology, with aspect ratios highly concentrated between 0.3 and 0.5, and the Weibull fit demonstrates high precision, indicating extremely high consistency and regularity in the particle morphology.

The parameters of Weibull distributions, mean values, and standard deviations for the aspect ratios of the different mono-gradations of four kinds of sands are shown in Table 2.

Particles with higher aspect ratios are relatively more difficult to move in soil because of the increased contact surfaces between particles, leading to higher internal friction and possibly higher porosity and greater compressibility, while particles with low aspect ratios are more likely to be compactly packed, resulting in increased soil compactness.

### 3.2. Distributions of Sphericities

**Figure 2:**
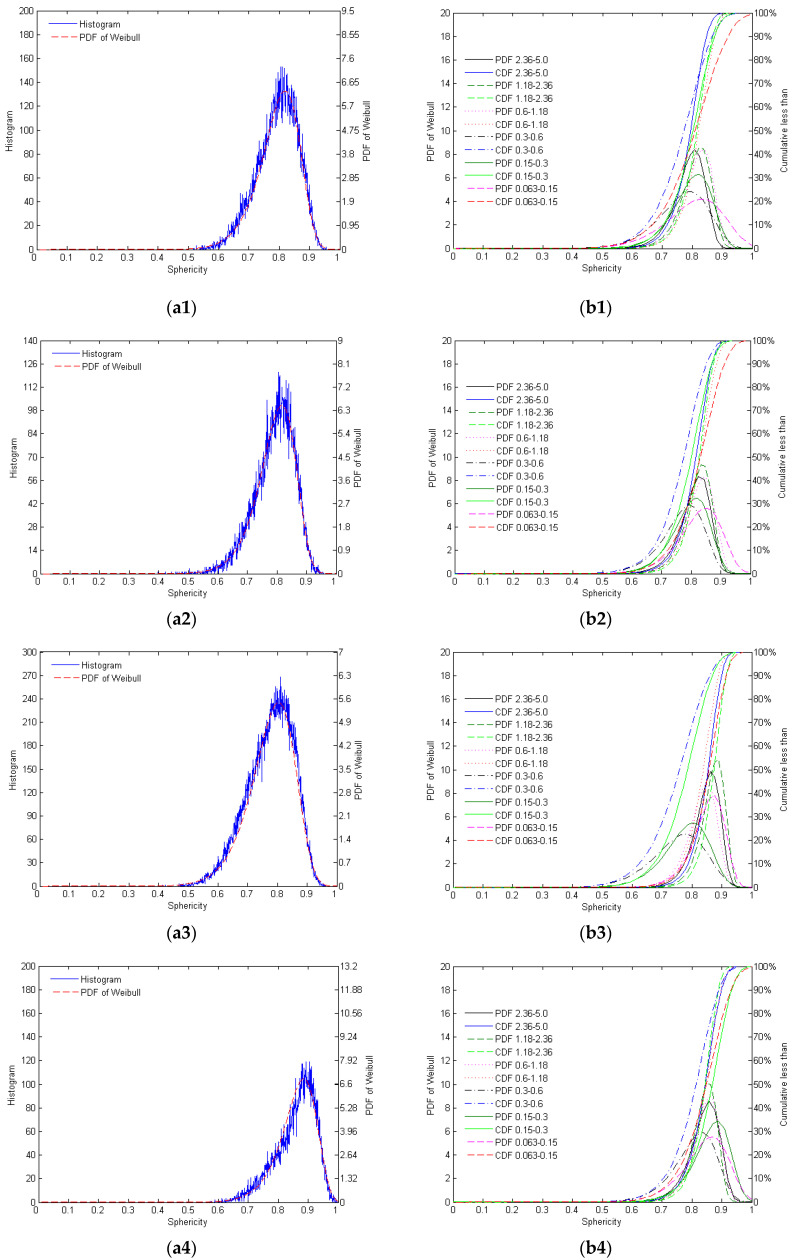
The distribution of sphericities of the different mono-gradations of AQS (**a1**,**b1**), ZRS (**a2**,**b2**), RQS (**a3**,**b3**), and LBS (**a4**,**b4**): (**a**) histogram for the mono-gradation ‘0.15–0.3’ and its Weibull PDF; (**b**) the PDF and CDF of Weibull distribution of different mono-gradations.

The distribution of sphericities of the different mono-gradations of crushed and natural sands is shown in Figure 2.

As shown in Figure 2 and Table 3, AQS particles exhibit relatively high sphericity, distributed between 0.75 and 0.9. However, due to the crushing process, some particles with lower sphericity are present, leading to greater variability in shape, particularly with irregularities in smaller particle size ranges. The sphericity of ZRS natural sand is concentrated between 0.75 and 0.9, with particles that are more rounded and exhibit higher consistency, while the proportion of low-sphericity particles is small. There is minimal variation in shape across different particle sizes, indicating a strong regularity in the morphology of natural sand. RQS shows a broader sphericity distribution, concentrated between 0.7 and 0.9. The particle morphology is relatively uniform, although some lower sphericity particles are present. Larger particles exhibit more regular shapes, with an overall moderate level of regularity. LBS particles display an extremely concentrated sphericity distribution, almost entirely between 0.8 and 1.0. The high sphericity across all particle size ranges suggests that the particle morphology is highly regular, approaching an ideal spherical shape.

### 3.3. Distributions of Ellipsoidal Degrees

Higher sphericity particles settle faster in fluids because the spherical shape reduces fluid resistance, making particle sinking more efficient [26]. Sand particles with high aspect ratios or low sphericity are prone to interlocking effects during packing, making it difficult for particles to slide or rearrange. Such particle shapes contribute to increased shear strength but may reduce packing efficiency. On the other hand, particles that are closer to spherical can pack more tightly, offering higher packing efficiency, which is suitable for applications requiring high-density filling.

**Figure 3:**
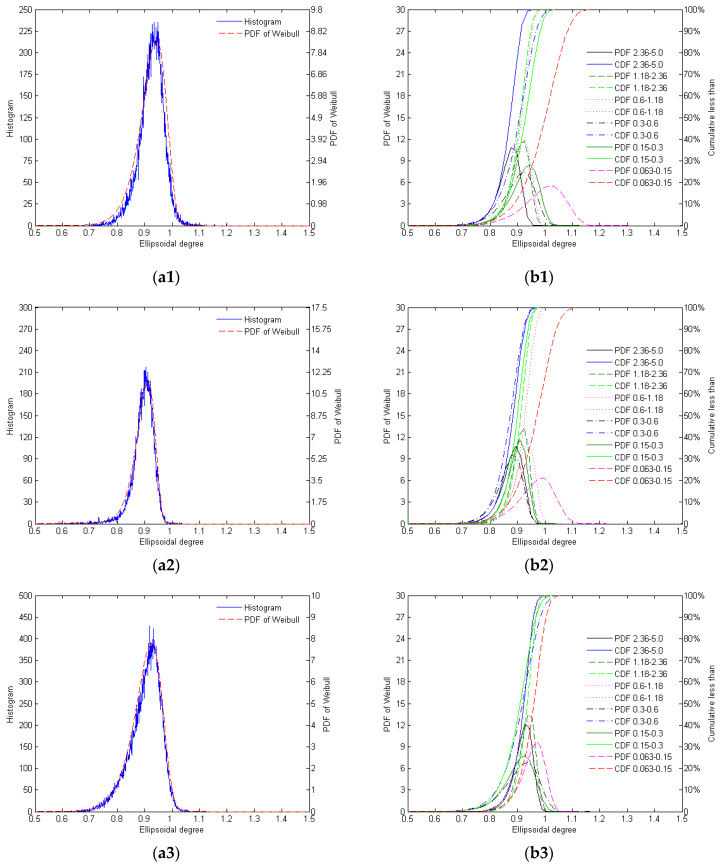

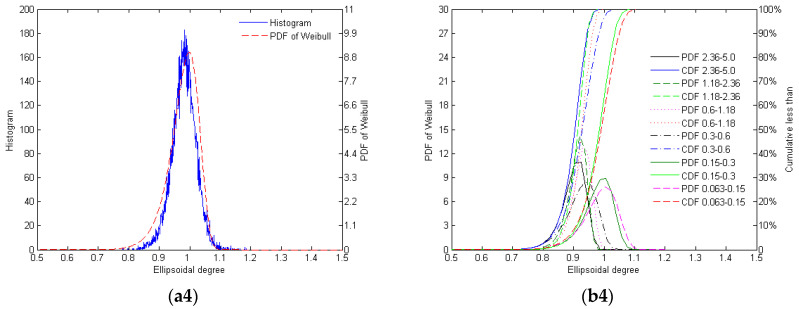
The distribution of ellipsoidal degrees of the different mono-gradations of AQS (**a1**,**b1**), ZRS (**a2**,**b2**), RQS (**a3**,**b3**), and LBS (**a4**,**b4**): (**a**) histogram for the mono-gradation ‘0.15–0.3’ and its Weibull PDF; (**b**) the PDF and CDF of Weibull distribution of different mono-gradations.

The distribution of ellipsoidal degrees of the different mono-gradations of crushed and natural sands is shown in Figure 3.

As shown in Figure 3 and Table 4, the ellipsoidality histograms of the four types of silica sand show a high degree of fit with the PDF of Weibull distribution. The ellipsoidality of AQS increases with particle size, with larger particles exhibiting more regular particle morphology, while smaller particles display greater variability in particle morphology. The particle morphology of ZRS is highly consistent, with ellipsoidality being concentrated across all particle size ranges and the particles approaching the shape of an ideal ellipsoid. RQS exhibits diverse particle morphology, with a wider distribution of ellipsoidality, particularly among smaller particles, which are less regular than natural sand. However, larger particles are closer to ellipsoidal in shape. The particle morphology of LBS is extremely consistent, with ellipsoidality being highly concentrated and particles across all size ranges closely resembling an ideal ellipsoid, demonstrating the highest consistency.

Among the four types, LBS shows the best consistency between the PDF of Weibull and the ellipsoidality, while ZRS natural sand also displays high regularity in particle morphology. RQS, due to its diversity of particles, has a slightly more dispersed morphology, whereas AQS particles become more regular as particle size increases.

## 4. Distributions of Micro-Size Indexes

Two microscale particle size indexes, specific surface and equivalent diameter, are prominently studied and evaluated, which show many differences with and more advantages than the macroscale particle size indexes, such as the median particle size by the screening test.

### 4.1. Specific Surfaces

#### 4.1.1. Distributions of Specific Surfaces

The differences in the equivalent diameters between the different materials with the same gradation, that is, the sub-size differences in the sub-gradation for different materials, may be the potential reasons for the variance in the macro-mechanics for different materials.

**Figure 4:**
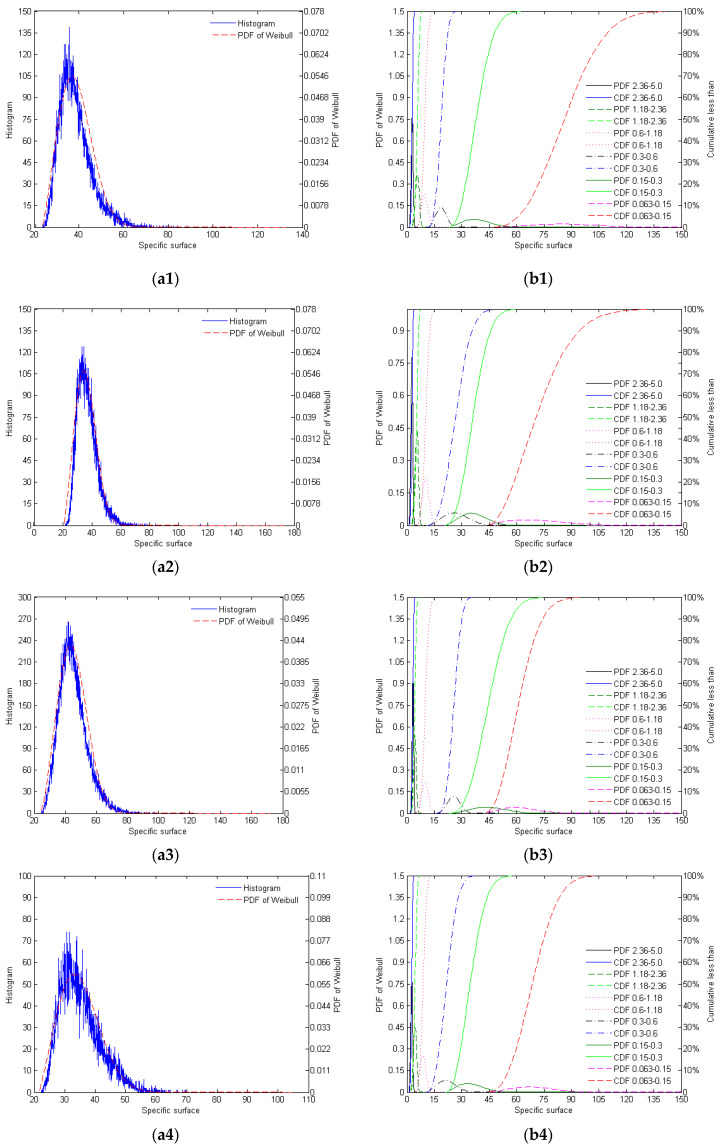
The distribution of specific surfaces of the different mono-gradations of AQS (**a1**,**b1**), ZRS (**a2**,**b2**), RQS (**a3**,**b3**), and LBS (**a4**,**b4**): (**a**) histogram for the mono-gradation ‘0.15–0.3’ and its Weibull PDF; (**b**) the PDF and CDF of Weibull distribution of different mono-gradations.

The distribution of specific surfaces of the different mono-gradations of crushed and natural sands is shown in Figure 4.

As shown in Figure 4 and Table 5, the specific surface area histograms of the four types of silica sand demonstrate a high degree of fit with the PDF of the Weibull distribution. AQS exhibits a relatively concentrated distribution of the specific surface area, but due to the randomness in particle morphology caused by the crushing process, there is still some morphological variation, resulting in a relatively broad distribution of the specific surface area. ZRS shows a more dispersed specific surface area distribution, with naturally formed particles, higher complexity, and a longer tail, indicating diverse particle morphology. RQS displays a more symmetrical and concentrated specific surface area distribution, with relatively consistent particle composition, and the fitting effect is good, suggesting a stable particle morphology. LBS has the most concentrated specific surface area distribution, with a highly regular particle morphology, showing the narrowest distribution range and an excellent fit, indicating a high degree of particle uniformity.

#### 4.1.2. Shape Variance with Specific Surface in a Mono-Gradation

**Figure 5:**
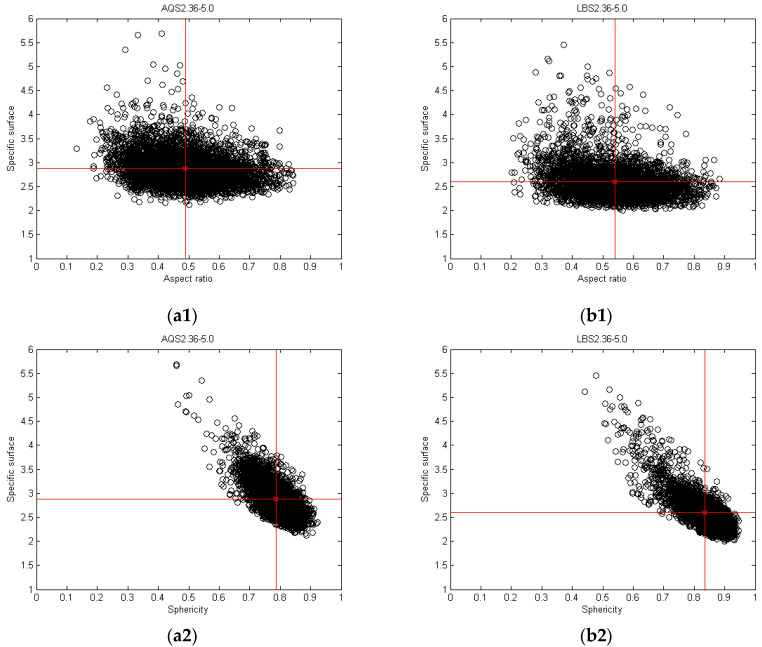

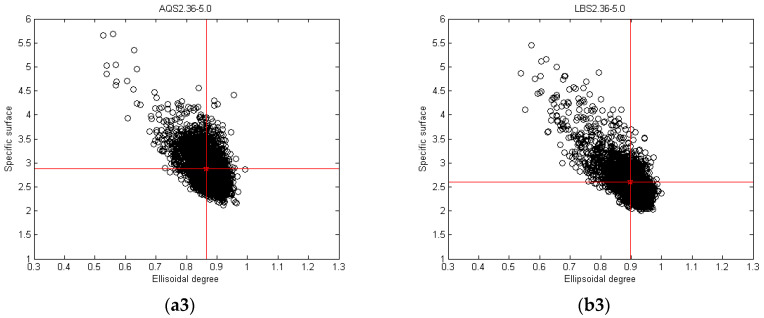
The relations between the particle shape and specific surface for mono-gradation: (**a1**–**a3**) AQS2.36–5.0 and (**b1**–**b3**) LBS2.36–5.0.

As shown in Figure 5, the distribution of LBS particles across various shape indices is more concentrated, indicating a higher shape regularity and a lower specific surface area. In contrast, AQS particles exhibit greater variations in shape, resulting in a broader range of the specific surface area distribution. Coarse particles have a much smaller specific surface area than fine particles, which means that coarse particles have a much smaller range of influence on scouring forces or weathering than fine particles in the same layer and similar environment. Consequently, for sphericity and ellipticity (the local angularity and roundness of particle compaction), fine particles are subject to greater scouring forces or weathering because their specific surface area is greater than that of coarse particles in the same layer and in similar environments. Particle shape characteristics vary with particle size and geological conditions of the material’s origin. For particles with the same single gradation, the relationship between the particle shape index and the equivalent diameter of the particles is not obvious or does not have a certain relationship. Instead, the particle shape index varies with the specific surface area of the particles. The larger the specific surface area, the smaller the particle shape index. The potential energy of a natural object is related to its own weight, and the contact between an object and its surroundings is related to its specific surface. Therefore, the specific surface area is the main index for measuring the morphological, physicochemical, and mechanical properties of particles.

#### 4.1.3. Difference in the Specific Surfaces for a Mono-Gradation of Difference Materials

**Figure 6:**
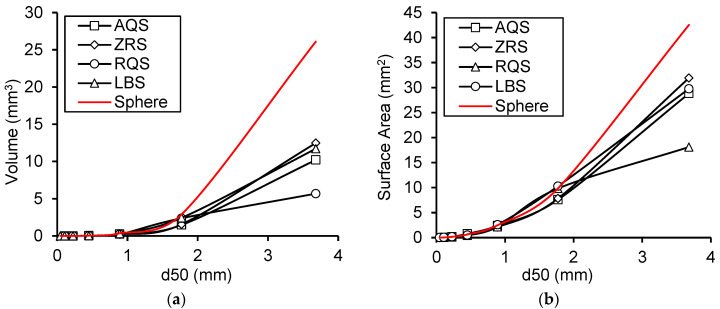

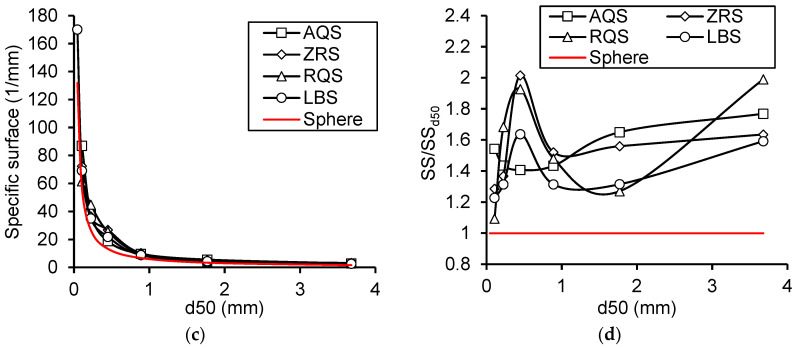
The differences in (**a**) the average particle volume, (**b**) the average surface area, (**c**) the specific surface, and (**d**) the relative specific surface of each mono-gradation between different kinds of materials.

As shown in Figure 6, both natural sand and crushed sand exhibit a higher specific surface area and relative specific surface area, especially within the small particle size range, which can likely be attributed to their surface roughness and irregular shapes. However, significant differences are observed under different gradations. The selection of different types of particle materials should be based on the specific application requirements. For instance, to enhance interparticle bonding strength, sands with higher specific surface areas should be prioritized, while sands with shapes closer to a sphere can be considered to improve interparticle packing performance.

### 4.2. Equivalent Diameters

#### 4.2.1. Distributions of Equivalent Diameters

**Figure 7:**
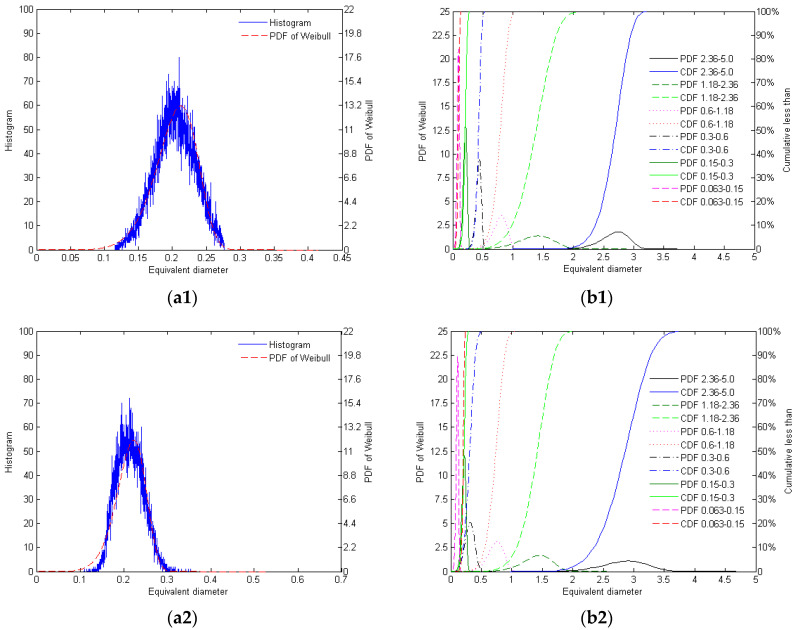

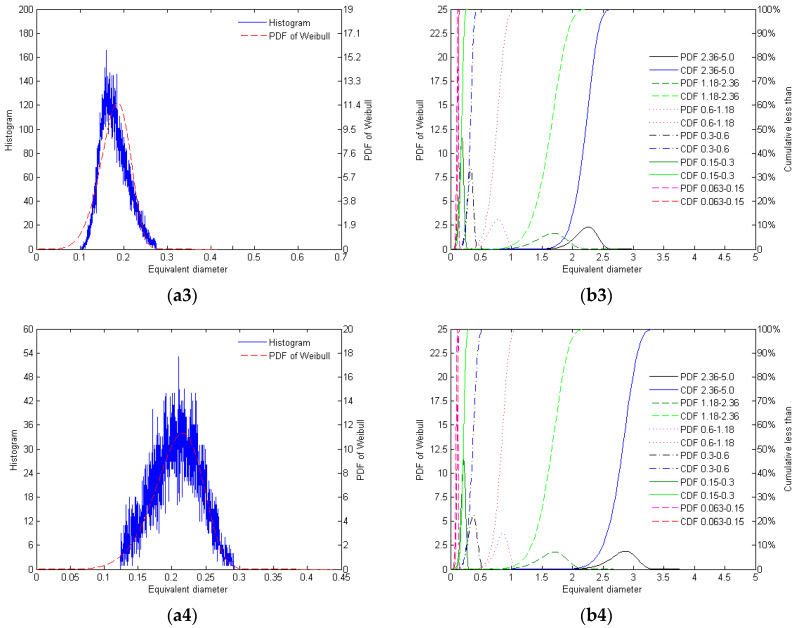
The distribution of equivalent diameters of the different mono-gradations of AQS (**a1**,**b1**), ZRS (**a2**,**b2**), RQS (**a3**,**b3**), and LBS (**a4**,**b4**): (**a**) histogram for the mono-gradation ‘0.15–0.3’ and its Weibull PDF; (**b**) the PDF and CDF of Weibull distribution of different mono-gradations.

From Figure 7, the distribution of equivalent diameters of the different mono-gradations of crushed and natural sands is shown in Figure 7.

As shown in Figure 7 and Table 6, the histograms of the equivalent diameters for the four types of silica sand, in the single gradation range of ‘0.15–0.3’, exhibit a high degree of fit with the PDF of the Weibull distribution. For AQS, its PDF shows significant variation in the distribution across different particle sizes. Smaller particle sizes (e.g., 0.063–0.15 mm) exhibit greater fluctuations in the equivalent diameter, whereas larger particle sizes (e.g., 2.36–5.0 mm) have a more concentrated distribution. The CDF curve indicates that as the equivalent diameter increases, the cumulative percentage of particles rises sharply, suggesting a relatively dispersed size distribution, particularly in the smaller particle size range. ZRS demonstrates the most uniform distribution of equivalent diameters, with a highly consistent particle morphology, reflecting the regularity and stability of the morphology of natural sand. The equivalent diameter distribution of RQS is relatively more dispersed, with diverse particle morphologies, and there is considerable variation in particle diameter, resulting in a relatively weaker regularity in the surface area and morphology. LBS, on the other hand, exhibits the most concentrated equivalent diameter distribution, with a regular particle morphology, showing the strongest consistency between the particle morphology and surface area, approaching an ideal particle shape.

#### 4.2.2. Shape Variance with Equivalent Diameter in a Mono-Gradation

**Figure 8:**
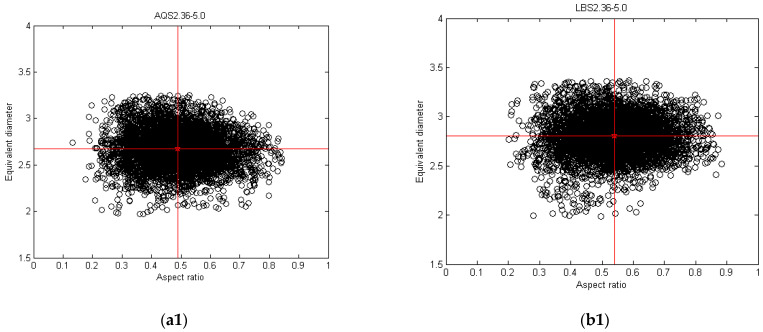

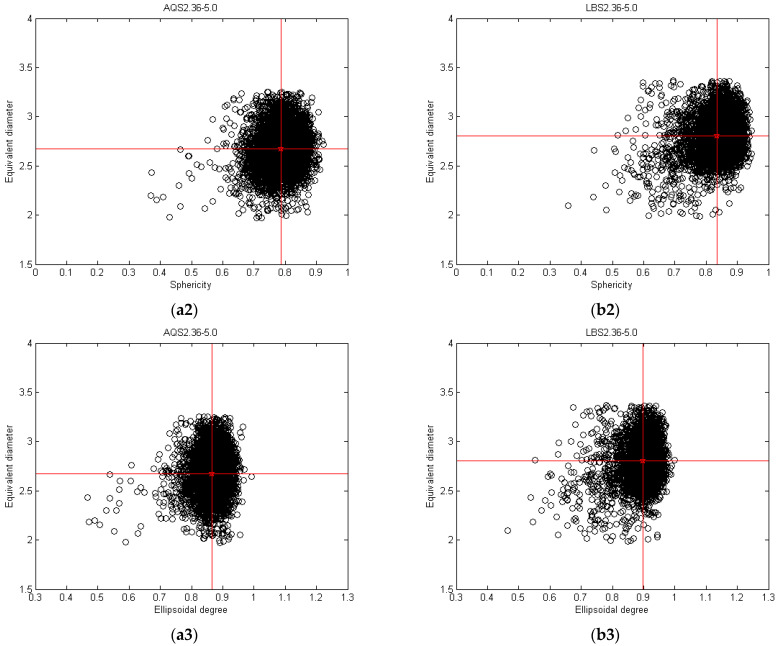
The relations between the particle shape and equivalent diameter for mono-gradation: (**a1**–**a3**) AQS2.36–5.0 and (**b1**–**b3**) LBS2.36–5.0.

Both AQS and LBS particles exhibit a relatively high regularity in shape. However, LBS particles demonstrate greater uniformity, with higher sphericity and ellipsoidality, whereas AQS particles show certain shape fluctuations, particularly in the small particle size range. As shown in Figure 8.

#### 4.2.3. Variance in Equivalent Diameters of Mono-Gradation of Difference Materials

**Figure 9:**
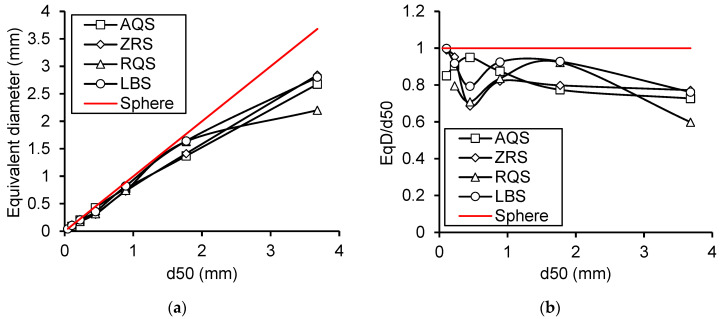
The differences in (**a**) the equivalent diameters and (**b**) the relative equivalent diameter of each mono-gradation between different kinds of materials.

Figure 9 shows the variance in the equivalent diameters of the same sub-mono-gradation between different kinds of materials. The differences in packing and macro-mechanics behaviour are caused by the variances in particle shapes and sub-mono-gradations of different materials with the same size gradation.

The equivalent diameters of particles are always smaller than the d50 of the mono-gradations.

The equivalent diameters of particles for the same mono-gradations of different materials always show a big difference.

Researchers always conduct comparison studies through the control variate method. Also, many studies of granular matter, especially geotechnics, perform comparison research on different types of materials by controlling the gradation of materials to be the same. However, the results of these studies sometimes suggest that the macro-scale behaviour of the materials may be affected by the sub-gradation, which is not measured or cannot be accurately measured. So, there is a need to propose an approach to characterize the accurate information of different gradations of different indexes. Then, the average value of equivalent diameters for all the particles using the CT imaging technique is better than the d50 of gradation or sub-gradation by a conventional sieving test.

## 5. Dependence of Particle Shape on Size

The dependence of 2D particle shape by QICPIC on the particle size of sands and the shape evolution of sands during compression was investigated by Altuhafi and Coop in an attempt to relate the soil behaviour to the nature of the microscopic particle damage during yielding.

The dependence of 3D true particle shape on particle size for the whole gradations of three natural and one crushed sands (0.063–5.0 mm) are investigated in this study. Zhou and Wang (2017) [26] investigated the relationship between size and 2D shape parameters using the QICPIC apparatus for hundreds of particles of LBS with coarse sizes of 0.6–5.0 mm. The limited number of particles with the 2D imaging information shows a large variability for natural sands. The significant effect of particle size on the distribution of the 3D shape parameters of sand fragments during size evolution under the mechanical crushing process was investigated by Zhao et al. (2015) [27]. This study comprehensively investigated hundreds of thousands of particles for four types of natural or crushed sands and gravels with sizes ranging from 0.063 to 5.0mm using the X-ray uCT facility. The dependences of 3D shape characteristics (aspect ratios, sphericity, ellipsoidal degree, and regularity) on particle size (ranges from 0.063 mm to 5.0 mm) are adequately investigated with uCT imaging for hundreds of thousands of particles of crushed and natural sands.

### 5.1. Variance in the Mean Values of Shape and Micro-Size Indexes

#### 5.1.1. Mean Value of Shape Indexes

The dependence of the 2D particle shape by QICPIC on the particle size of sands was investigated by Altuhafi and Coop. This study comprehensively investigates the 3D particle shape characteristics of hundreds of thousands of particles for four kinds of natural or crushed sands and fine gravels with sizes ranging from 0.063 to 5.0 mm using 3D imaging technology and X-ray micro-computerized tomography.

**Figure 10:**
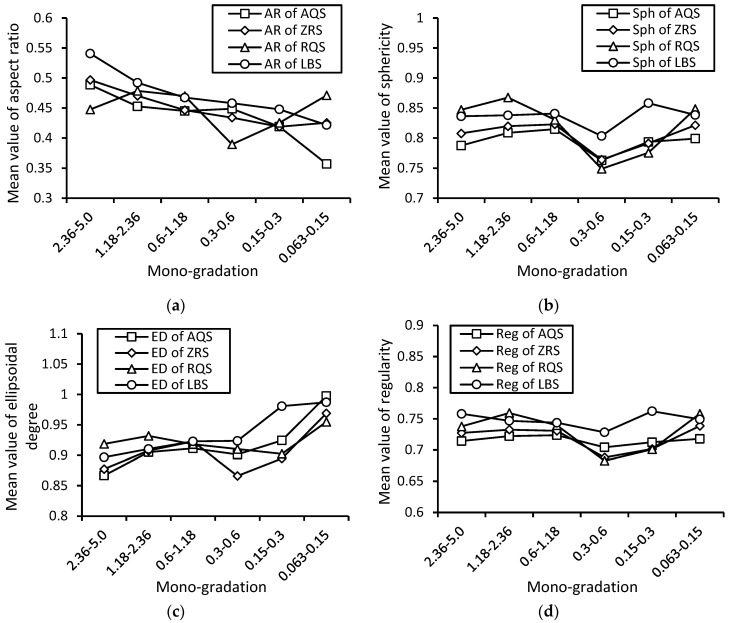
Dependences of 3D particle shape indexes, (**a**) aspect ratios, (**b**) sphericity, (**c**) ellipsoidal degree, (**d**) regularity, on particle sizes for crushed and natural sands. Dependences of 3D particle shape indexes, (**a**) aspect ratios, (**b**) sphericity, (**c**) ellipsoidal degree, (**d**) regularity, on particle sizes for crushed and natural sands.

The dependences of particle shapes on size for crushed and natural sands are shown in Figure 10. The trends of the dependences of particle shapes on sizes for natural and crushed sands are similar, which may reveal that the formation mechanism of the natural sands is the same as the crushed sands. Both evolved and developed from large grains to small grains. However, the variances in shape for crushed sands are bigger than the variances in natural sands, which may be because the natural sand has been scoured and weathered more uniformly.

The aspect ratio is the higher level of particle shape characteristics, which means the overall anisotropy of the particle form. The approximate decreasing trends of the dependences of the aspect ratio on particle sizes for natural sands and crushed sands are similar. It is known that the coarse grains are always on the surface for moving granular packings, such as gravel river beds, to play the role of an “armoured” layer due to the segregation phenomenon of granular matter [2]. Thus, most of the coarse grains are always in the “armoured” layer, and they suffer much greater scouring force or weathering, which will lead to the anisotropy of coarse grains becoming smaller; that is, the aspect ratio (overall isotropy of the form of particulates) of coarse grains becomes larger.

The sphericity and the ellipsoidal degree are the middle level of shape characteristics; they mean the local angularity and roundness of particle compactness. The “S”-typed trends of the dependences of the sphericity and the ellipsoidal degree on sizes for natural sands and crushed sands are similar. Regarding the ranges from ‘2.36~5.0’ (fine gravel) to ‘0.6~1.18’ (coarse sands), the shape indexes increase with the decrease in particle sizes. Regarding the ranges from ‘0.6~1.18’ (coarse sands) to ‘0.3~0.6’ (medium sands), the shape indexes decrease with the decrease in particle sizes. Regarding the ranges from ‘0.3~0.6’ (medium sands) to ‘0.063~0.15’ (fine sands), the shape indexes increase with the decrease in particle sizes. It can be observed that the gravel and the medium sands are always more angular (local compactness of particulates) than the coarse sands and fine sands. The reasons may be the combined effects of the following factors:

(a) Most of the coarse grains are always in the “armoured” layer due to segregation, and they suffer much greater scouring force or weathering; this stronger natural environment will lead to the sphericity and ellipsoidal degree (local angularity and roundness of the compactness of particulates) of coarse grains becoming larger.
(b) However, the specific surface of the coarse grains is much smaller than that of fine grains, which means that the extent of the effects of scouring force or weathering for coarse grains is much smaller than that of fine grains if they are in the same layer and under the similar environment. Thus, for the sphericity and ellipsoidal degree (local angularity and roundness of the compactness of particulates), the fine particulates suffered greater scouring force or weathering due to their bigger specific surface than the coarse grains in the same layer and a similar environment.

The regularity of the particulate is the average value of the aspect ratio, sphericity, and ellipsoidal degree. Thus, the relationship between the regularity and particle size is influenced by the balance of the dependences of the three basic shape indexes, each with a different level of influence on the morphology and particle size.

#### 5.1.2. Mean Value of Micro-Size Indexes

**Figure 11:**
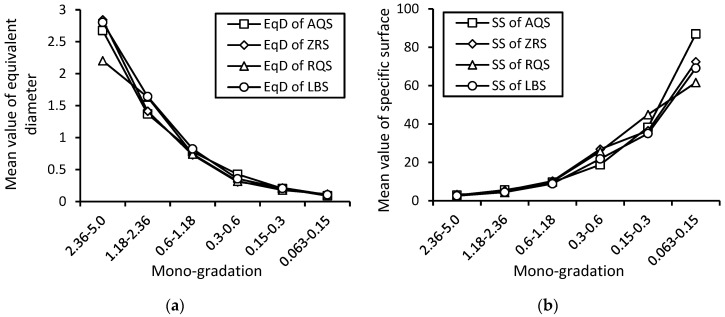
Dependences of micro-size indexes, (**a**) equivalent diameter and (**b**) specific surface, on macro-particle sizes for crushed and natural sands.

The dependences of micro-size indexes on macro-particle sizes are shown in Figure 11. With the decrease in particle size of mono-gradations, the mean values of equivalent diameter decrease at different degrees for different kinds of sands, while the mean values of the specific surface increase at different degrees for different kinds of sands.

### 5.2. Variance in the Standard Deviations of Shape and Micro-Size Indexes

#### 5.2.1. Standard Deviations of Shape Indexes

**Figure 12:**
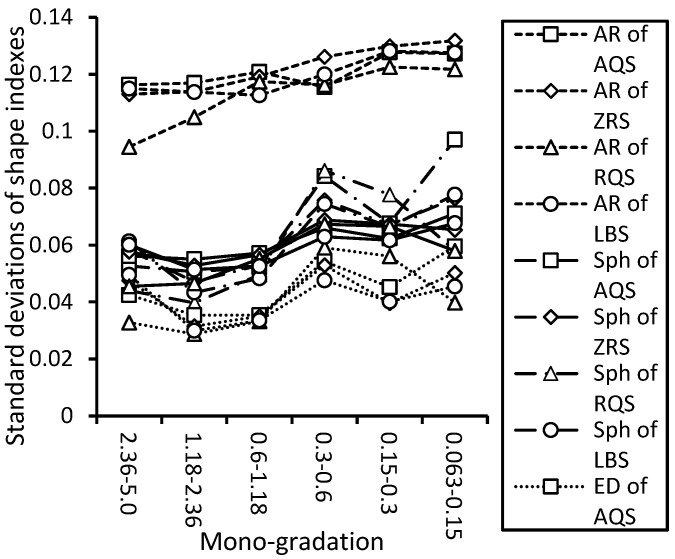
The standard deviation of particle shape indices of each mono-gradation for crushed and natural sands.

The values of the standard deviations (fluctuation of values) of shape indexes roughly increase with the decreases in the particle sizes. Also, the larger the values of shape indexes, the smaller the standard deviations are, as shown in Figure 12.

#### 5.2.2. Standard Deviation of Micro-Size Indexes

For the fine sand gradation, 0.063–0.15 mm, of the crushed sands AQS, the standard deviation of the specific surface is quite larger than the natural sands RQS and LBS. The fine sand gradation, 0.063–0.15 mm, of the natural sands ZRS should be similar to the crushed fragments.

**Figure 13:**
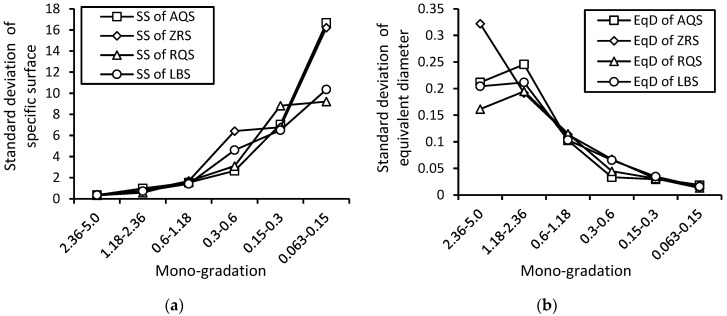
Variance in the standard deviations of micro-size indexes, (**a**) specific surfaces, (**b**) equivalent diameter, of particles of each mono-gradation for crushed and natural sands.

From Figure 13, coarse gravel particles show much more variance in the equivalent diameter than the particles of sand. Also, coarse sands show much more variance in the equivalent diameter than fine sands. The strong variance in the specific surfaces with particle sizes suggests that the fine particles sometimes play an important role in the whole system of a wide-graded granular assembly. This provides an important topic to explore in the study of material properties after particle filling.

## 6. Conclusions

The distribution patterns of the particle shape and size characteristics for crushed and natural sands are amply investigated by X-ray computed microtomography (uCT) and contrastively analyzed through statistical analysis. A 3D shape index named the ellipsoidal degree is defined to evaluate the roundness of a particle with an explicit calculation. The dependences of 3D shape characteristics (aspect ratios, sphericity, and ellipsoidal degree) on particle size (ranges from 0.063 mm to 5.0 mm) are adequately investigated with uCT imaging for hundreds of thousands of particles of crushed and natural sands. The study focuses on examining and evaluating two microscale particle size indicators—the specific surface and equivalent diameter. The conclusions are as follows:

- The distribution pattern of shape and micro-size characteristics of particles of the mechanical crushing sands is similar to that of particles of the natural sands, which both have a skewed normal distribution, which can be expressed by the Weibull distribution function. The trends of the dependences of particle shapes on sizes for natural and crushed sands are similar, which may reveal that the formation mechanism of natural sands is the same as crushed sands. However, the variances in shape for crushed sands are bigger than the variances in natural sands, which may be because the natural sand has been scoured and weathered more uniformly.
- The size and shape characteristics of soil particles reflect their formation history. The dependence of 3D particle shape characteristics on the particle size is evident, especially regarding the specific surface area of microscopic features. The aspect ratio, representing the overall particle anisotropy, decreases with particle size for both natural and crushed sands. The reasons may be due to the combined effects of the following factors:
  (1) Most of the coarse grains are always in the “armoured” layer due to segregation, and they suffer stronger scouring force or weathering; this stronger natural environment will lead to the sphericity and ellipsoidal degree (local angularity and roundness of the compactness of particulates) of coarse grains becoming larger.
  (2) However, the specific surface of the coarse grains is much smaller than that of fine grains, which means the extent of the effects of scouring force or weathering for coarse grains is much smaller than that of fine grains if they are in the same layer and under the similar environment. Thus, for the sphericity and ellipsoidal degree (local angularity and roundness of the compactness of particulates), the fine particulates suffered greater scouring force or weathering due to their bigger specific surface than the coarse grains in the same layer and a similar environment.
- The particle shape characteristics vary with the particle size and the geological conditions of the material source. For particles with the same mono-gradation, the particle shape index is not obvious or has no definite relation with the particle’s equivalent diameter. However, the particle shape index changes with the particle’s specific surface. The larger the specific surface is, the smaller the particle shape index is. The potential energy of a natural object is related to its own weight, while the contact of an object with its surroundings is related to its specific surface. Therefore, the specific surface is the main index to measure the morphological properties, physicochemical properties, and mechanical properties of particles.
- The morphology of natural particles exhibits high variability and a wide range of changes. Therefore, studying particle morphology alone is insufficient; the number of particles must also be considered. In particular, the shape characteristics of crushed sand are more dispersed.

The conclusions of this study provide a micro-scale explanation for particle sedimentation behaviour and offer robust data references for future discrete element simulations to further analyze the impact of micro-scale material properties on overall performance. Future research will focus on exploring the influence of characteristic scales of micro-scale 3D particle shape descriptors (such as aspect ratio and sphericity) on macroscopic behaviour (e.g., packing and mechanical behaviour), aiming to provide valuable insights for developing higher-performance materials in the field of materials science.

## Figures and Tables

**Table 1:** Comparison of the ellipsoidal degrees and the traditional roundness for the typical 3D geometries.

Types of Geometry	Descriptions	Proposed Ellipsoidal Degree	Roundness by Wadell
Sphere	All types	1	1
Ellipsoid	All types	1	<1
Triangular pyramid	Equilateral types	Specific	Uncertain
Cube	All types	Specific	Uncertain

**Table 2:** Parameters of Weibull distributions, mean values, and standard deviations for the aspect ratios of the different mono-gradations of crushed and natural sands.

Materials	Mono-Gradations (mm)	k	λ	θ	Mean	Standard Deviation
AQS	2.36–5.0	0.534	4.563	0.000	0.489	0.116
	1.18–2.36	0.498	4.157	0.000	0.453	0.117
	0.6–1.18	0.491	3.979	0.000	0.445	0.121
	0.3–0.6	0.493	4.162	0.000	0.449	0.115
	0.15–0.3	0.465	3.571	0.000	0.419	0.128
	0.063–0.15	0.400	2.963	0.000	0.357	0.127
ZRS	2.36–5.0	0.542	4.743	0.000	0.497	0.113
	1.18–2.36	0.515	4.454	0.000	0.470	0.114
	0.6–1.18	0.492	4.058	0.000	0.447	0.119
	0.3–0.6	0.480	3.740	0.000	0.434	0.126
	0.15–0.3	0.466	3.533	0.000	0.419	0.130
	0.063–0.15	0.473	3.490	0.000	0.425	0.132
RQS	2.36–5.0	0.486	5.049	0.000	0.448	0.095
	1.18–2.36	0.521	4.820	0.000	0.479	0.105
	0.6–1.18	0.516	4.315	0.000	0.470	0.117
	0.3–0.6	0.432	3.561	0.000	0.390	0.116
	0.15–0.3	0.471	3.739	0.000	0.425	0.123
	0.063–0.15	0.518	4.183	0.000	0.471	0.122
LBS	2.36–5.0	0.587	5.218	0.000	0.541	0.115
	1.18–2.36	0.537	4.684	0.000	0.492	0.114
	0.6–1.18	0.512	4.443	0.000	0.467	0.113
	0.3–0.6	0.504	4.132	0.000	0.458	0.120
	0.15–0.3	0.495	3.819	0.000	0.448	0.128
	0.063–0.15	0.468	3.533	0.000	0.422	0.128

**Table 3:** Parameters of Weibull distributions, mean values, and standard deviations for the sphericities of the different mono-gradations of crushed and natural sands.

Materials	Mono-Gradations (mm)	k	λ	θ	Mean	Standard Deviation
AQS	2.36–5.0	0.924	21.025	−0.113	0.787	0.053
	1.18–2.36	0.831	19.484	0.000	0.809	0.051
	0.6–1.18	0.838	19.205	0.000	0.815	0.052
	0.3–0.6	0.800	10.501	0.000	0.763	0.084
	0.15–0.3	0.824	14.075	0.000	0.794	0.068
	0.063–0.15	0.842	9.547	0.000	0.799	0.097
ZRS	2.36–5.0	1.403	32.238	−0.571	0.808	0.058
	1.18–2.36	0.840	21.492	0.000	0.820	0.047
	0.6–1.18	0.846	19.107	0.000	0.823	0.053
	0.3–0.6	1.377	22.055	−0.579	0.764	0.076
	0.15–0.3	0.829	14.610	−0.009	0.791	0.067
	0.063–0.15	0.855	13.047	0.000	0.821	0.076
RQS	2.36–5.0	0.867	23.307	0.000	0.847	0.044
	1.18–2.36	0.885	26.425	0.000	0.867	0.040
	0.6–1.18	0.852	20.617	0.000	0.830	0.050
	0.3–0.6	0.787	9.610	0.000	0.749	0.086
	0.15–0.3	0.809	12.042	0.000	0.776	0.078
	0.063–0.15	1.336	28.663	−0.462	0.848	0.058
LBS	2.36–5.0	1.502	35.202	−0.641	0.836	0.061
	1.18–2.36	0.857	23.779	0.000	0.838	0.043
	0.6–1.18	0.862	21.003	0.000	0.841	0.048
	0.3–0.6	1.123	18.054	−0.286	0.803	0.074
	0.15–0.3	1.314	24.681	−0.427	0.858	0.066
	0.063–0.15	0.873	13.067	0.000	0.838	0.078

**Table 4:** Parameters of Weibull distributions, mean values, and standard deviations for the ellipsoidal degrees of the different mono-gradations of crushed and natural sands.

Materials	Mono-Gradations (mm)	k	λ	θ	Mean	Standard Deviation
AQS	2.36–5.0	1.418	42.892	−0.534	0.867	0.042
	1.18–2.36	0.921	29.784	0.000	0.905	0.035
	0.6–1.18	0.927	29.772	0.000	0.911	0.035
	0.3–0.6	0.926	18.609	0.000	0.901	0.054
	0.15–0.3	0.945	21.663	0.000	0.924	0.045
	0.063–0.15	1.025	15.486	0.000	0.997	0.060
ZRS	2.36–5.0	1.346	39.553	−0.450	0.877	0.048
	1.18–2.36	0.922	33.523	0.000	0.907	0.031
	0.6–1.18	0.938	29.601	0.000	0.923	0.035
	0.3–0.6	1.450	37.948	−0.562	0.866	0.053
	0.15–0.3	1.074	33.969	−0.162	0.895	0.040
	0.063–0.15	0.992	17.142	0.000	0.969	0.050
RQS	2.36–5.0	0.934	31.333	0.000	0.919	0.033
	1.18–2.36	0.945	35.520	0.000	0.932	0.029
	0.6–1.18	0.933	32.254	0.000	0.918	0.033
	0.3–0.6	0.937	17.505	0.000	0.910	0.059
	0.15–0.3	0.927	19.537	0.000	0.902	0.056
	0.063–0.15	0.973	25.955	0.000	0.955	0.040
LBS	2.36–5.0	1.452	44.989	−0.536	0.897	0.050
	1.18–2.36	0.924	35.787	0.000	0.910	0.030
	0.6–1.18	0.938	32.996	0.000	0.923	0.034
	0.3–0.6	0.945	21.800	0.000	0.924	0.048
	0.15–0.3	1.000	24.521	0.000	0.981	0.040
	0.063–0.15	1.008	21.539	0.000	0.987	0.045

**Table 5:** Parameters of Weibull distributions, mean values, and standard deviations for the specific surfaces of the different mono-gradations of crushed and natural sands.

Materials	Mono-Gradations (mm)	k	λ	θ	Mean	Standard Deviation
AQS	2.36–5.0	1.908	3.892	1.119	2.884	0.363
	1.18–2.36	4.005	3.859	1.956	5.593	0.988
	0.6–1.18	4.872	2.871	5.308	9.668	1.537
	0.3–0.6	8.626	3.087	11.036	18.754	2.652
	0.15–0.3	17.403	2.311	22.893	38.284	7.040
	0.063–0.15	45.739	2.583	46.356	86.937	16.673
ZRS	2.36–5.0	2.140	4.398	0.691	2.666	0.406
	1.18–2.36	3.727	4.387	1.860	5.288	0.759
	0.6–1.18	5.609	3.148	5.227	10.253	1.691
	0.3–0.6	17.729	2.594	11.131	26.880	6.417
	0.15–0.3	17.733	2.442	20.775	36.486	6.777
	0.063–0.15	32.399	1.889	43.853	72.458	16.210
RQS	2.36–5.0	1.875	4.801	1.509	3.246	0.331
	1.18–2.36	2.787	3.994	1.746	4.306	0.585
	0.6–1.18	5.411	3.046	5.136	9.979	1.670
	0.3–0.6	10.747	3.213	16.056	25.704	3.105
	0.15–0.3	24.111	2.567	23.501	44.904	8.823
	0.063–0.15	21.630	2.179	42.482	61.599	9.213
LBS	2.36–5.0	1.729	3.588	1.007	2.596	0.363
	1.18–2.36	2.965	3.469	1.765	4.458	0.729
	0.6–1.18	4.401	2.834	4.930	8.858	1.419
	0.3–0.6	12.826	2.596	10.438	21.824	4.616
	0.15–0.3	15.431	2.231	21.406	35.037	6.497
	0.063–0.15	28.589	2.559	43.745	69.122	10.358

**Table 6:** Parameters of Weibull distributions, mean values, and standard deviations for the equivalent diameters of the different mono-gradations of crushed and natural sands.

Materials	Mono-Gradations (mm)	k	λ	θ	Mean	Standard Deviation
AQS	2.36–5.0	1.796	8.724	0.973	2.675	0.212
	1.18–2.36	1.476	5.489	0.000	1.371	0.246
	0.6–1.18	0.825	7.899	0.000	0.779	0.103
	0.3–0.6	0.444	11.367	0.000	0.427	0.033
	0.15–0.3	0.216	7.671	0.000	0.204	0.030
	0.063–0.15	0.098	5.506	0.000	0.091	0.019
ZRS	2.36–5.0	2.003	5.909	0.976	2.841	0.322
	1.18–2.36	1.499	6.773	0.000	1.413	0.192
	0.6–1.18	0.781	6.584	0.000	0.731	0.114
	0.3–0.6	0.336	4.553	0.000	0.309	0.067
	0.15–0.3	0.228	7.374	0.000	0.214	0.031
	0.063–0.15	0.173	10.453	−0.059	0.106	0.019
RQS	2.36–5.0	1.627	10.247	0.648	2.202	0.161
	1.18–2.36	1.722	7.756	0.000	1.633	0.195
	0.6–1.18	0.795	6.675	0.000	0.744	0.115
	0.3–0.6	0.339	7.633	0.000	0.319	0.045
	0.15–0.3	0.192	5.976	0.000	0.179	0.031
	0.063–0.15	0.123	10.345	0.000	0.117	0.013
LBS	2.36–5.0	1.906	9.699	0.987	2.803	0.204
	1.18–2.36	1.734	8.454	0.000	1.642	0.212
	0.6–1.18	0.868	8.833	0.000	0.823	0.103
	0.3–0.6	0.385	5.807	0.000	0.357	0.066
	0.15–0.3	0.221	6.735	0.000	0.207	0.035
	0.063–0.15	0.113	7.691	0.000	0.106	0.016

## Data Availability

The original contributions presented in the study are included in the article, further inquiries can be directed to the corresponding author.
